# Effect of feedback on delaying deterioration in quality of compressions during 2 minutes of continuous chest compressions: a randomized manikin study investigating performance with and without feedback

**DOI:** 10.1186/1757-7241-20-16

**Published:** 2012-02-28

**Authors:** Tobias Stenbjerg Lyngeraa, Peter Buhl Hjortrup, Nille Birk Wulff, Theis Aagaard, Anne Lippert

**Affiliations:** 1 Danish Institute for Medical Simulation, Copenhagen University Hospital, Herlev Hospital, Copenhagen, Denmark

**Keywords:** Cardiopulmonary resuscitation (CPR), Basic life support (BLS), Advanced life support (ALS), Cardiac arrest, Resuscitation

## Abstract

**Background:**

Good quality basic life support (BLS) improves outcome following cardiac arrest. As BLS performance deteriorates over time we performed a parallel group, superiority study to investigate the effect of feedback on quality of chest compression with the hypothesis that feedback delays deterioration of quality of compressions.

**Methods:**

Participants attending a national one-day conference on cardiac arrest and CPR in Denmark were randomized to perform single-rescuer BLS with (n = 26) or without verbal and visual feedback (n = 28) on a manikin using a ZOLL AED plus. Data were analyzed using Rescuenet Code Review. Blinding of participants was not possible, but allocation concealment was performed. Primary outcome was the proportion of delivered compressions within target depth compared over a 2-minute period within the groups and between the groups. Secondary outcome was the proportion of delivered compressions within target rate compared over a 2-minute period within the groups and between the groups. Performance variables for 30-second intervals were analyzed and compared.

**Results:**

24 (92%) and 23 (82%) had CPR experience in the group with and without feedback respectively. 14 (54%) were CPR instructors in the feedback group and 18 (64%) in the group without feedback. Data from 26 and 28 participants were analyzed respectively. Although median values for proportion of delivered compressions within target depth were higher in the feedback group (0-30 s: 54.0%; 30-60 s: 88.0%; 60-90 s: 72.6%; 90-120 s: 87.0%), no significant difference was found when compared to without feedback (0-30 s: 19.6%; 30-60 s: 33.1%; 60-90 s: 44.5%; 90-120 s: 32.7%) and no significant deteriorations over time were found within the groups. In the feedback group a significant improvement was found in the proportion of delivered compressions below target depth when the subsequent intervals were compared to the first 30 seconds (0-30 s: 3.9%; 30-60 s: 0.0%; 60-90 s: 0.0%; 90-120 s: 0.0%). Significant differences were not found in secondary outcome and in other performance variables between the groups and over time

**Conclusions:**

Quality of CPR was maintained during 2 minutes of continuous compressions regardless of feedback in a group of trained rescuers.

## Background

Quality chest compressions along with defibrillation are the cornerstones of cardiopulmonary resuscitation (CPR) and improves outcome of cardiac arrest [1]. In the European Resuscitation Council (ERC) 2010 guidelines for Basic Life Support (BLS) further emphasis has been placed on the quality of CPR and the use of adjuncts to assist the rescuer in performing BLS [2]. These recommendations of adjuncts are based on studies showing that quality increases with feedback [3,4]. Furthermore the ERC 2010 guidelines recommend that compressions-only CPR be performed if rescuers are not trained in performing ventilations. Continuous compressions are also recommended in the Advanced Life Support (ALS) algorithm, when an endotracheal tube has been placed and inflations and compressions are performed independently [5]. A major concern with uninterrupted chest compressions is deterioration in quality of CPR. The influence of fatigue on chest compressions has been studied and results differ widely. While most results indicate a significant decrease in compression depth and/or rate within 1 minute [6,7], others show a slower decrease or no decrease in 10 minutes [8]. A recent study investigated the effects of fatigue on quality of chest compressions delivered during continuous chest compressions in-hospital and found that depth of compressions decayed after 90 seconds even though feedback adjuncts were used [9]. These results stem from trained cardiac arrest response teams in-hospital. No comparison with CPR without feedback was done in that study so the effect of feedback in delaying a decrease in quality of the delivery of continuous compressions over time remains to be elucidated. The advent of new guidelines and recommendations that deeper and more frequent compressions are needed might make deterioration in quality of delivered CPR over time due to fatigue an even more important factor in quality CPR. The aim of the study was to investigate the effect of feedback on quality of chest compression with the hypothesis that feedback delays deterioration of quality of compressions over time.

## Methods

This study was performed as a parallel superiority study to evaluate the effect of feedback on deterioration in quality of compressions over time. During a one-day conference on cardiac arrest and CPR, participants of the conference were randomly selected by sealed envelopes to participate in the study. 30 envelopes for each group were placed by a conference official before participants arrived, hidden underneath 60 of approximately 150 non-numbered chairs in the lecture hall. All conference participants were eligible. A 1:1 allocation ratio was used and informed consent was obtained. Hence the participants belonged to either a group that performed feedback guided chest compressions on a manikin (AMBU, Ballerup, Denmark) with an AED (ZOLL AED plus, ZOLL Medical Corporation, Chelmsford, MA) or a group that performed chest compressions without feedback on a manikin with an AED (ZOLL AED plus, ZOLL Medical Corporation, Chelmsford, MA). Blinding was not possible, but allocation concealment was upheld until participants were in the room with the manikin.The feedback group was instructed in the visual and verbal feedback features. In the group not receiving feedback the speakers were disconnected and the display was covered. All participants were instructed to perform 2 minutes of uninterrupted chest compressions on the AMBU manikin, which was already fitted with AED pads. The AMBU manikins in both groups were placed on the floor to provide a hard surface and CPR was performed with the rescuer kneeling beside the manikin.

### AED

The ZOLL AED Plus is fitted with CPR-D-pads including a "puck" with an accelerometer and force transducer that is placed on the patient's sternum and registers the rate and depth of chest compressions. Voice prompts and visual messages on the monitor screen are triggered when chest compressions are measured to deviate from guidelines or are interrupted. A real-time bar graph in the display guides the rescuer with real-time feedback on depth. A built-in metronome guides the rescuer to optimal compression rate by delivering sound beats that initially are close to the frequency of chest compressions delivered and increases or decreases in order to guide the rescuer's own rate towards the recommended compression rate. The AED records the information concerning the chest compressions which can be extracted to a computer and further analysed using Rescuenet Code review (ZOLL Medical Corporation, Chelmsford, MA). This displays a tabular summary of CPR performance variables within a specified time interval, determining the absolute number of compressions above, in or below the target zone as well as the percentage of total compressions. Below target is defined as compressions between 0 and 4.99 cm. In target is defined as compressions between 5.0 and 6.0 cm and above target as compressions 6.01 cm and deeper. Compression rate is displayed in the same manner where in target is defined as between 100-120 compressions per minute.

### AMBU manikins

The manikins were adjusted to medium stiffness. This was done by aligning the top part of the notch with the top of the marker for "medium" stiffness.

### Sample size

Sample size was calculated on the basis of wanting to detect a mean difference of 25% with alpha = 0.05 and a beta = 0.8. Deterioration of 25% (SD 27,2) has been found in the proportion of delivered compressions within correct depth from minute 1 to minute 2 [10]. Detection of this deterioration would require 10 participants in each group. When investigating differences in the proportion of delivered compressions within correct depth between groups performing CPR with and without feedback, a standard deviation of SD 33 has been found [11]. Thus to detect a difference of 25% between would require a sample size of 56. Taking potential dropouts or technical difficulties into consideration we therefore aimed to include 60 participants.

### Data analysis

Data on the performed chest compressions were gathered from the AEDs and analysed. The 2-minute periods were segmented into 30-second intervals and analysed using RescueNet Code Review. The 30-second intervals were summarised as the proportion of compressions being above target, in target or below target for depth as well as for rate.

### Statistical analysis

A difference between performance variables for 30-second intervals within each group were identified using Friedman's repeated measurements on ranks, ANOVA, and Student-Newman-Keuls test was applied for multiple pair-wise comparisons when appropriate (Sigmaplot 11, Systat software Inc, Chicago, IL). Comparisons between performance variables for 30-second intervals in the feedback and non-feedback group were analysed using Student's unpaired *t*-test when data were normally distributed; otherwise Mann-Whitney Rank sum test was used. Differences were considered significant when *P *< 0.05.

### Outcome

Primary outcome was the proportion of delivered compressions within target depth compared over a 2-minute period within the groups and between the groups. Secondary outcome was the proportion of delivered compressions within target rate compared over a 2-minute period within the groups and between the groups.

## Results

**Figure 1:**
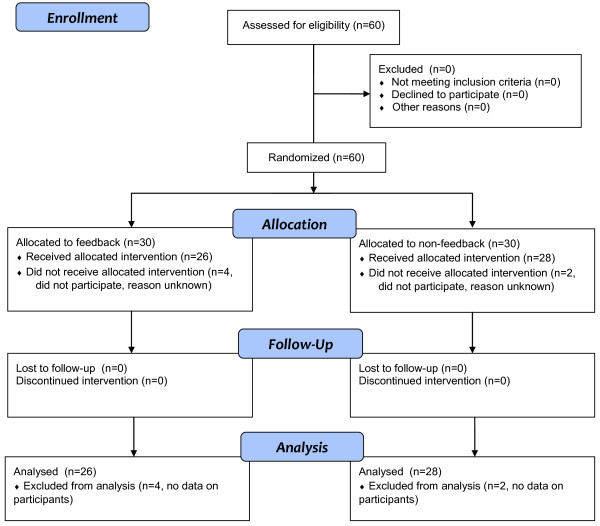
**CONSORT 2010 Flow Diagram**.

Data from 54 participants were analyzed in the study, see Figure 1. The age, gender and previous CPR experience of the subjects in the two groups were well matched and distributed as shown in Table 1.

**Table 1:** Characteristics of participants

	with feedback (n = 26)	without feedback (n = 28)
Mean age - years (range)	45 (24-66)	44 (25-64)

Males:females	18:8	20:8

Has CPR experience*	24	23

BLS or generic CPR instructor	14	18

*Either BLS or ALS certified provider/instructor, generic CPR instructor or paramedic/nurse/doctor with CPR experience.

### With feedback

Performance variables for the group receiving feedback are shown in table 2. No significant difference in the proportions of delivered compressions within depth was found over time. This was also the case for the proportions of delivered compressions with correct compression rate over time. A significant improvement was seen between the proportions of delivered compressions below target in the first 30-second interval compared to the subsequent intervals.

**Table 2:** Performance variables for chest compressions performed with feedback

Variable	0 to 29.9 s	30 s to 59.9 s	60 s to 89.9 s	90 s to 120 s
Compressions above target (%)	20.4(0.00-92.4)	2.04(0.00-98.0)	2.00(0.00-100)	0.00(0.00-88.98)

Compressions within target (%)	54.0(5.06-82.9)	88.0(2.00-99.0)	72.6(0.00-99.0)	87.0(2.02-100)

Compressions below target (%)	3.92(0.85-13.2)	0.00(0.00-1.00)*	0.00(0.00-2.73)*	0.00(0.00-1.04)*

Mean compression depth (cm)	5.99 SD 0.85	6.00 SD 0.82	5.85 SD 0.78	5.79 SD 0.79

Rate above target (%)	0.00 (0.00-2.91)	0.00 (0.00-0.00)	0 .00 (0.00-0.00)	0.00 (0.00-0.00)

Rate within target (%)	64.0(38.8-82.4)	66.0(56.0-84.3)	62.0 (52.0-85.3)	62.5 (51.6-81.5)

Rate below target (%)	20.0 (2.90-47.0)	30.0 (2.78-40.0)	34.0 (2.77-41.0)	28.9 (0.91-43.8)

Mean compression rate (min^-1^)	102.6 SD 9.6	103.2 SD 9.4	102.7 SD 9.2	103.1 SD 9.7

values are depicted as medians and interquartile range in parenthesis. Mean depth and rate as means with standard deviation (SD).*p < 0.05 compared to the first interval

### Without feedback

Performance variables for the group not receiving feedback are shown in table 3. There were no significant changes in primary and secondary outcome measures as well as other performance variables over time.

**Table 3:** Performance variables for chest compressions performed without feedback

Variable	0 to 29.9 s	30 s to 59.9 s	60 s to 89.9 s	90 s to 120 s
Compressions above target (%)	73.2 (0.00-95.6)	53.4 (0.00-100)	23.5 (0.00-100)	29.6 (0.00-99.5)

Compressions within target (%)	19.6 (2.26-60.2)	33.1 (0.00-81.9)	44.5 (0.00-74.7)	32.7 (0.00-83.6)

Compressions below target (%)	0.90 (0.00-3.76)	0.00 (0.00-0.00)	0.00 (0.00-26.9)	0.00 (0.00-6.35)

Mean compression depth (cm)	5.94 SD 0.77	5.92 SD 0.77	5.87 SD 0.77	5.88 SD 0.77

Rate above target (%)	0.00 (0.00-39.9)	0.0 (0.00-33.3)	0.00 (0.00-13.1)	0.00 (0.00-9.33)

Rate within target (%)	61.0 (26.4-95.9)	58.0 (4.98-100)	48.5 (5.24-100)	47.4 (5.95-99.6)

Rate below target (%)	1.85 (1.72-36.8)	0.00 (0.00-49.8)	0.00 (0.00-67.6)	0.84 (0.00-68.4)

Mean compression rate (min^-1^)	108.8 SD 10.7	108.6 SD 12.1	107.6 SD 12.0	107.1 SD 12.2

values are depicted as medians with interquartile range in parenthesis. Mean depth and rate as means with standard deviation (SD). *p < 0.05 compared to the first (0-29.9 s) interval

### With feedback versus without feedback

Statistical comparisons between the two groups showed no significant differences.

## Discussion

No statistically significant differences were found in our primary outcome measures. Comparisons over time within the groups did not reveal deterioration over time in any of the groups. This is in contrast with other studies that have shown deterioration in quality of CPR after 1 minute using even fewer participants [6,7]. While it has been shown that feedback significantly improves the quality of CPR [11] our study does not support this finding. However, comparisons between groups may have been under-powered as our sample size was slightly smaller than calculated to be necessary a priori and our data suggest a trend with the feedback group achieving higher proportions of delivered compressions within correct depth than the non-feedback. The participants in our study were highly motivated and skilled as many of the participants were CPR instructors. This, in turn, may explain why we did not find deterioration over time in any of the groups. This would also reasonably lessen the positive effect of feedback between the groups explaining why no significant results were found. We propose that the effect should be examined in the setting where CPR is performed by persons not experienced in delivering CPR because feedback could potentially have an effect on delaying deterioration in quality of performance under these circumstances. Our study suggests that in the highly motivated and skilled rescuer quality CPR can be maintained for a 2-minute interval regardless of feedback. And perhaps even longer as it was shown earlier that trained paramedics can maintain delivery of quality CPR for 10 minutes [8]. The results from our study are in good accordance with a recently published study where only 5 out of 19 experienced paramedics, who performed ALS according to Norwegian guidelines, showed decay in quality of chest compression below guidelines during a 12-minute period without CPR feedback. Only one showed deterioration within the first 2 minutes [12]. Norwegian guidelines differ from ERC guidelines both regarding recommended depth of compressions and interval between defibrillations making direct comparisons difficult [13]. Further studies are needed to investigate whether longer CPR periods can be performed without deterioration in quality during CPR with and without feedback and to what extent a potential deterioration in quality is influenced by feedback. A limitation of our study is that our eligibility and randomization procedure was flawed according to CONSORT criteria as randomization occurred prior to consent retrieval. As it was only 4 (13%) and 2 (7%) randomized participants in the two groups respectively that did not participate we do not believe it had an impact on the study findings. Furthermore, we have only investigated one 2-minute period of continuous CPR on a hard surface. The deterioration in quality of CPR will perhaps be more explicit during prolonged periods of CPR only interrupted by defibrillation or under circumstances where chest compressions are performed on a suboptimal surface. Yet performing CPR on a manikin in a bed or on the floor without feedback has been shown to cause deterioration in quality of compressions with no significant difference between the two situations [14]. Thus further studies are needed to examine whether feedback has a more pronounced effect during longer resuscitation periods and when CPR is performed on a softer surface e.g. a bed.

## Conclusion

Results from our study showed that quality of CPR was maintained during 2 minutes of continuous compressions regardless of feedback in a group of trained rescuers.

## Competing interests

The authors declare that they have no competing interests. The study was performed at a conference held by Medidane Hjertevagt ApS. TSL, PBH, TA and NBW received an unrestricted contribution of 2000 Dkr in total by Medidane Hjertevagt ApS. Medidane Hjertevagt ApS made contributions to study design but authors had final say. Medidane Hjertevagt ApS had no role in data collection and analysis, decision to publish, or preparation of the manuscript.

## Authors' contributions

TSL, PBH and AL contributed to conception and design. TSL, PBH, TA and NBW helped in execution of the study and data collection. TSL performed data analysis, and drafting of the manuscript. All authors revised the manuscript for important intellectual content and approved the final manuscript.
